# A Retrospective Study Analyzing Sexually Transmitted Infections and Bacterial Vaginosis

**DOI:** 10.7759/cureus.93969

**Published:** 2025-10-06

**Authors:** Pariyat Jain, Raj Rathee, Kris Vijay

**Affiliations:** 1 Obstetrics and Gynecology, Estrella Women's Health Center, Phoenix, USA; 2 Cardiology, University of Arizona College of Medicine - Phoenix, Phoenix, USA

**Keywords:** bacterial vaginosis (bv), prevalence, prevention initiatives, public health education, retrospective cohort, sexually transmitted infections (stis)

## Abstract

Background: Sexually transmitted infections (STIs) are often caused by bacteria, viruses, parasites, and fungi. Some STIs include herpes, human papillomavirus (HPV), Chlamydia trachomatis, Neisseria gonorrhoeae, and Trichomonas vaginalis, whose risk increases significantly in the presence of bacterial vaginosis (BV), an infection that creates a bacterial imbalance in the body. Despite this relationship, there is limited understanding of this correlation across varying demographics, such as age and ethnicity.

Methods: In a retrospective cohort of 175 symptomatic specimens, using results from the Aptima Multitest Swab Specimen Collection Kit and the ThinPrep Pap Test to assess bacterial vaginosis, and the Aptima Urine Specimen Collection Kit and HealthLink TransPorter Sterile Transport Swab to assess STIs, this study evaluated the correlation between both infections.

Results: The median age was 22 years (range 18-62). BV was positive in 14.3% of African Americans, 61.1% of Hispanics, 3.4% of Pacific Islanders, and 21% of White participants. STI was positive in 44%, BV in 76.6%, and either STI or BV in 100% of cases. Individuals in the childbearing age group had a significantly higher prevalence of STI or BV (73.7%) compared to perimenopausal (17.1%) or menopausal (9.1%) age groups (chi-square 130.09; p<0.0001). There was no significant relationship between ethnicity and positive STI or BV (chi-square 19.8; p=0.7). The likelihood of having both STIs and BV was highly significant compared to having only one infection (chi-square 72.03; p<0.0001).

Conclusions: Our findings suggest that both STIs and BV are significantly associated with a higher likelihood of concomitant infection. Women of childbearing age have a higher susceptibility to developing both STIs and BV. This study underscores the need for prevention strategies and early screening in high-risk demographics to improve patients’ reproductive health.

## Introduction

Sexually transmitted infections (STIs) and bacterial vaginosis (BV) often co-occur and are common reproductive health concerns affecting individuals, particularly women, worldwide. Prominent STIs include Chlamydia trachomatis, Neisseria gonorrhoeae, herpes, human immunodeficiency virus (HIV), and Trichomonas vaginalis. These infections are caused by the transmission of bacteria, viruses, or parasites through sexual activity [1]. BV is a vaginal infection caused by an imbalance of bacteria in a woman’s genital tract. Although they are separate conditions, STIs and BV are closely linked, as symptoms often overlap and the presence of one infection increases susceptibility to the other [2]. Common overlapping symptoms include vaginal discharge, increased vaginal pH, odor, itching, and bleeding [3]. The relationship between BV and STIs is complex, and the impact of demographic factors such as ethnicity and age on this relationship continues to be actively studied. Previous research has shown the presence and severity of BV and STIs across ethnic groups, including African Americans and Hispanics [4]. These infections also occur across different life stages, including childbearing, perimenopausal, and menopausal women, influencing the manifestation and risk of STIs and BV.

What are sexually transmitted infections?

STIs are primarily transmitted through sexual contact, though some are spread solely via skin-to-skin contact. They are caused by bacteria, viruses, or parasites and disproportionately affect women worldwide. Over twenty types of STIs exist; this study focuses on Chlamydia trachomatis, Neisseria gonorrhoeae, herpes, HIV, and Trichomonas vaginalis [5]. Other common STIs include human papillomavirus (HPV), syphilis, and hepatitis B, which remain prevalent globally. The global burden of STIs has increased in recent years, with millions affected [5]. Marginalized populations often face limited access to preventive resources, including condoms, sexual health education, and awareness of STIs, contributing to higher infection rates and highlighting the importance of studies in these populations.

Bacterial vaginosis and its relationship to sexually transmitted infections

BV occurs when the vaginal bacterial balance is disrupted, most commonly during the reproductive years. Normally, the vagina maintains a balance of “good” and “bad” bacteria; in BV, anaerobic bacteria increase, disrupting this balance. Hormonal changes and sexual activity increase susceptibility, particularly in women [6]. Untreated BV can adversely affect sexual, physical, and mental health and increase the risk of acquiring STIs such as Chlamydia, Neisseria gonorrhoeae, herpes, and HIV [6]. Despite these risks, the relationship between BV and STIs is understudied. Symptoms often overlap, including abnormal discharge, vaginal odor, irritation, and pain during urination. Without proper diagnosis and treatment, co-occurring infections may go undetected, leading to adverse outcomes.

Significance of patient demographics

This study analyzed specimens across demographic factors, including age and ethnicity. Patients were categorized as childbearing (ages 20-40), perimenopausal (ages 41-50), and menopausal (ages 51 and older). Age categorization is important because hormonal fluctuations, sexual activity, and immune system responsiveness influence susceptibility to STIs and BV, aiding in early detection and prevention [7]. Ethnic groups included Pacific Islander, White, African American, and Hispanic. Considering ethnicity is crucial for identifying socioeconomic or biological factors contributing to disparities in reproductive health. Higher prevalence within certain groups allows targeted prevention and education, highlighting differences in healthcare access and potential economic disparities. Demographics help interpret infection patterns and identify populations at greater risk.

Objectives

The aim of this study is to examine the prevalence and co-occurrence of STIs and BV across ethnic and age groups to identify potential relationships and populations at highest risk. Specifically, it assesses rates of STIs and BV among Hispanic, African American, White, and other populations. Additionally, it examines the relationship between age groups (childbearing, perimenopausal, and menopausal) and susceptibility to STIs and BV. The study also explores how demographic variables such as age and ethnicity influence the co-occurrence of STIs and BV and highlights potential disparities in infection prevalence and access to screening or care.

## Materials and methods

A total of 175 vaginal specimens were collected from 175 women using the Aptima Multitest Swab Specimen Collection Kit and the ThinPrep Pap Test for testing BV, and the Aptima Urine Specimen Collection Kit and HealthLink TransPorter Sterile Transport Swab to test STIs and BV. The retrospective cohort study occurred in a clinic between March 1 and June 30, 2024. For inclusion/exclusion criteria, patients were categorized into childbearing (20-40 years), perimenopausal (41-50 years), and menopausal (51+ years) age groups. While this study did not include a formal negative control or comparison group, its primary objective was exploratory: to assess patterns of STIs and BV prevalence across demographic variables within the study population. This design provided insights into associations and potential disparities within a real-world clinical sample, particularly among underrepresented groups. The inclusion of a control group in future research could enhance interpretability by providing a clearer baseline. Despite this limitation, the study contributes important foundational data and correlations that can inform more rigorous, controlled investigations.

To determine the required sample size and perform a power analysis for the association between age groups and STIs, a chi-square test for independence was used, as it is appropriate for two categorical variables. Similarly, for analyzing the likelihood of having both STIs and BV, a chi-square test for independence was applied. A conventional effect size, such as Cohen's w=0.3 (medium effect) or w=0.5 (large effect), was used for calculations. The key inputs for this power analysis were a statistical test: chi-square test for independence, significance level alpha=0.05, desired power (1-β)=80% (0.80), effect size: Cohen's w for the chi-square test, and degrees of freedom=2. This calculation yielded a required total sample size of n=85 for the medium effect size and a smaller sample size for a large effect size (Cohen's w=0.5) [8]. A medium effect size was used to ensure robust statistical evidence. Assuming 85 subjects in each group (STIs and BV), the expected total was 170 subjects. The study analyzed 175 subjects to account for potential missing data.

Participants’ charts were analyzed for ethnicity, which was recorded as Pacific Islander, African American, White, or Hispanic. Data were abstracted from patient charts to provide characteristics, vitals, and outcomes. Patients with positive screenings for STIs and BV were further analyzed, and the specific type of STI was recorded. Patient demographics, including age and ethnicity, were obtained from their charts. To maintain patient confidentiality, only necessary information was collected and analyzed, including age group, ethnicity, and STI or BV status. After analysis, all charts and findings excluded identifying information such as names or exact dates of birth to protect privacy and comply with HIPAA guidelines. All previous data were deleted from the clinic’s servers. The collected data were processed into charts to explore correlations between various STIs and BV across demographics. Chi-square tests for association were conducted to analyze relationships between age groups, ethnicity, STIs, and BV. Statistical analysis was performed using Medcalc version 23.2 (MedCalc Software Ltd., Ostend, Belgium).

## Results

Table 1 presents the baseline characteristics of the 175 specimens analyzed in the study, including ethnicity, age, and clinical findings. The data show that the majority of participants were Hispanic, 107 (61.1%), followed by White, 37 (21%), African American, 25 (14.3%), and Pacific Islander, 6 (3.4%). The median age was 33 years, with a range of 18-62 years. Notably, 134 (76.6%) of the participants tested positive for BV, 77 (44%) were positive for an STI, and all 175 (100%) participants were either BV or STI positive. These figures underscore the high prevalence of infections in the sample population and highlight the demographic skew toward Hispanic individuals.

**Table 1:** Baseline data

Median age (range)	33 (range 18-62)
BV positive	134 (76.6%)
STI positive	77 (44%)
BV or STI positive	175 (100%)
African American	25 (14.3%)
Hispanics	107 (61.1%)
Pacific Islanders	6 (3.4%)
White	37 (21%)

**Figure 1:**
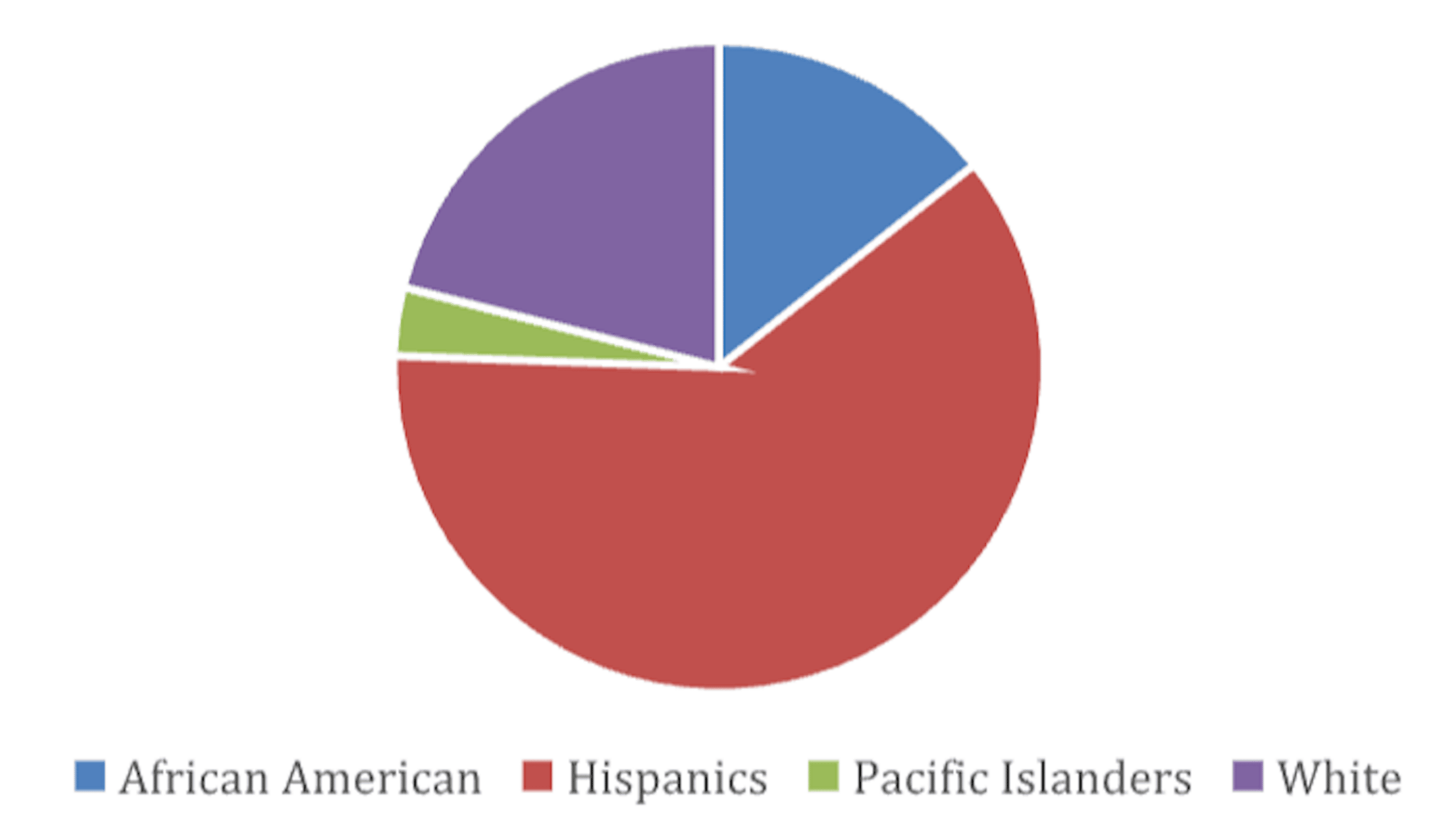
Prevalence of ethnicity

Figure 1 illustrates the ethnic distribution of the 175 specimens analyzed in the study. The largest group represented is Hispanic, 107 (61.1%), followed by White, 37 (21%), African American, 25 (14.3%), and Pacific Islander, 6 (3.4%), as shown in the pie chart.

**Figure 2:**
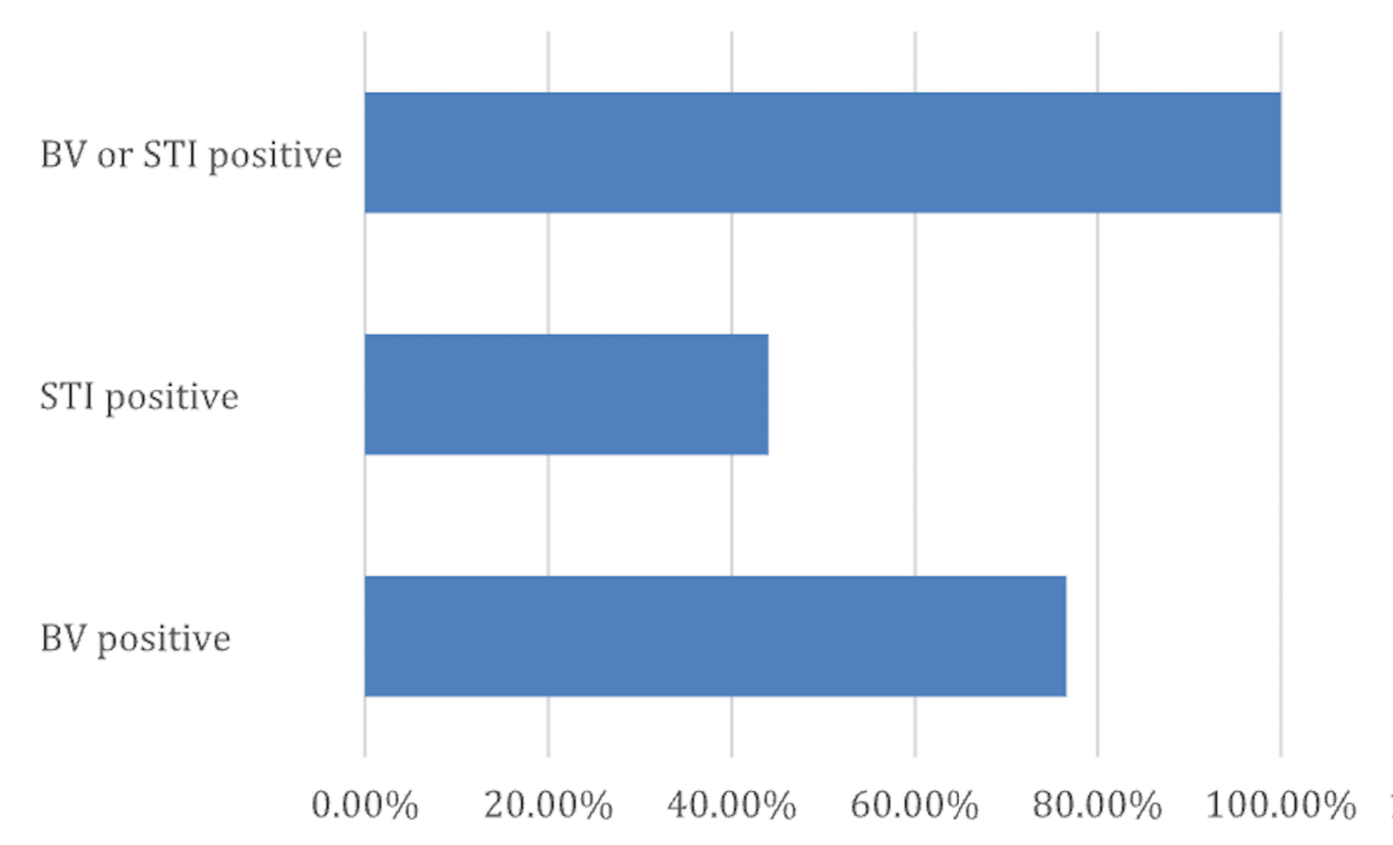
Prevalence of STIs and BV BV positive: 76.6%; STI positive: 44%; BV or STI positive: 100%. STIs, sexually transmitted infections; BV, bacterial vaginosis

Figure 2 highlights the clinical prevalence of BV and STIs in the study population. A significant majority, 134 (76.6%), tested positive for BV, while 77 (44%) were found to have at least one STI. Importantly, all individuals, 175 (100%), tested positive for either BV or an STI, underscoring the high burden of infection in this cohort. These findings are consistent with the study’s targeted selection criteria and may reflect broader public health concerns in the population studied.

**Figure 3:**
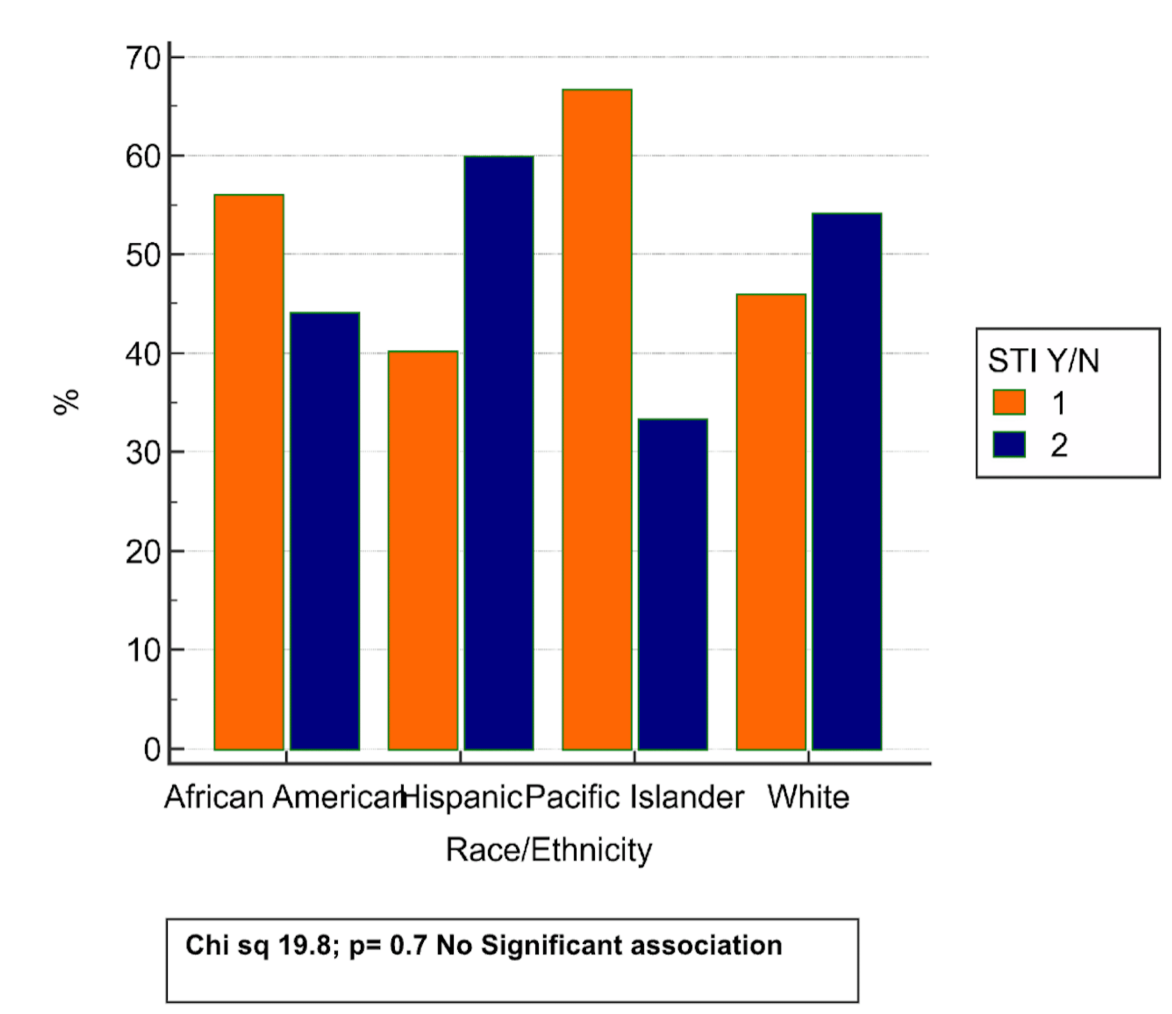
Prevalence of STIs in varying ethnicities STIs, sexually transmitted infections

Figure 3 illustrates the distribution of STI positivity across different racial and ethnic groups within the study population. While variation in STI prevalence is observed, for example, Pacific Islanders showed the highest percentage of STI-positive individuals (approximately 68%) and Hispanics the lowest (around 40%), these differences were not statistically significant. The chi-square test yielded a value of 19.8 with a p-value of 0.7, indicating no significant association between race or ethnicity and STI status. These findings suggest that STI prevalence in this cohort may not be strongly influenced by racial or ethnic background alone and may instead reflect other underlying factors.

Table 2 outlines the distribution of STI or BV positivity across different age groups within the sample population. The childbearing group accounted for the highest proportion of positive cases, 125 (73.7%), followed by the perimenopausal group, 30 (17.1%), and the menopausal group, 20 (9.1%). The chi-square test revealed a statistically significant association between age group and infection status (χ²=130.09, p<0.0001), indicating that reproductive age is a strong predictor of infection prevalence in this cohort. These findings underscore the importance of targeting screening and prevention efforts toward individuals in their childbearing years.

**Table 2:** Positive STIs and BV cases across different age groups within 175 specimens BV, bacterial vaginosis; STI, sexually transmitted infection

Age group	Proportion of STIs and BV	Significance
Childbearing	73.7%	
Perimenopausal	17.1%	Chi-square: 130.09
Menopausal	9.1%	P<0.0001

Table 3 explores the association between BV and specific types of STIs. Among BV-positive individuals, the overwhelming majority had no STI detected, though small proportions tested positive for HPV, Chlamydia trachomatis, and other infections at lower frequencies. In contrast, BV-negative individuals showed a higher diversity of STI types, with HPV and Chlamydia trachomatis being the most prevalent. The chi-square test yielded a value of 72.03 with a p-value <0.0001, indicating a statistically significant relationship between BV status and the type of STI. These findings suggest distinct patterns of infection co-occurrence and may have implications for diagnostic and treatment strategies.

**Table 3:** Relationship between STIs and BV in percentages 0=no STIs, 1=Human papillomavirus, 2=Chlamydia trachomatis, 3=Herpes, 4=Gonorrhea trachomatis, 5=Trichomonas vaginalis. STIs, sexually transmitted infections; BV, bacterial vaginosis

	0	Human papillomavirus	Chlamydia trachomatis	Herpes	Gonorrhea trachomatis	Trichomonas vaginalis
BV: Yes	73.1	12.7	7.5	1.5	3.7	1.5
BV: No	0	63.4	17.1	7.3	7.3	4.9
Chi-square: 72.03; p<0.0001						

**Figure 4:**
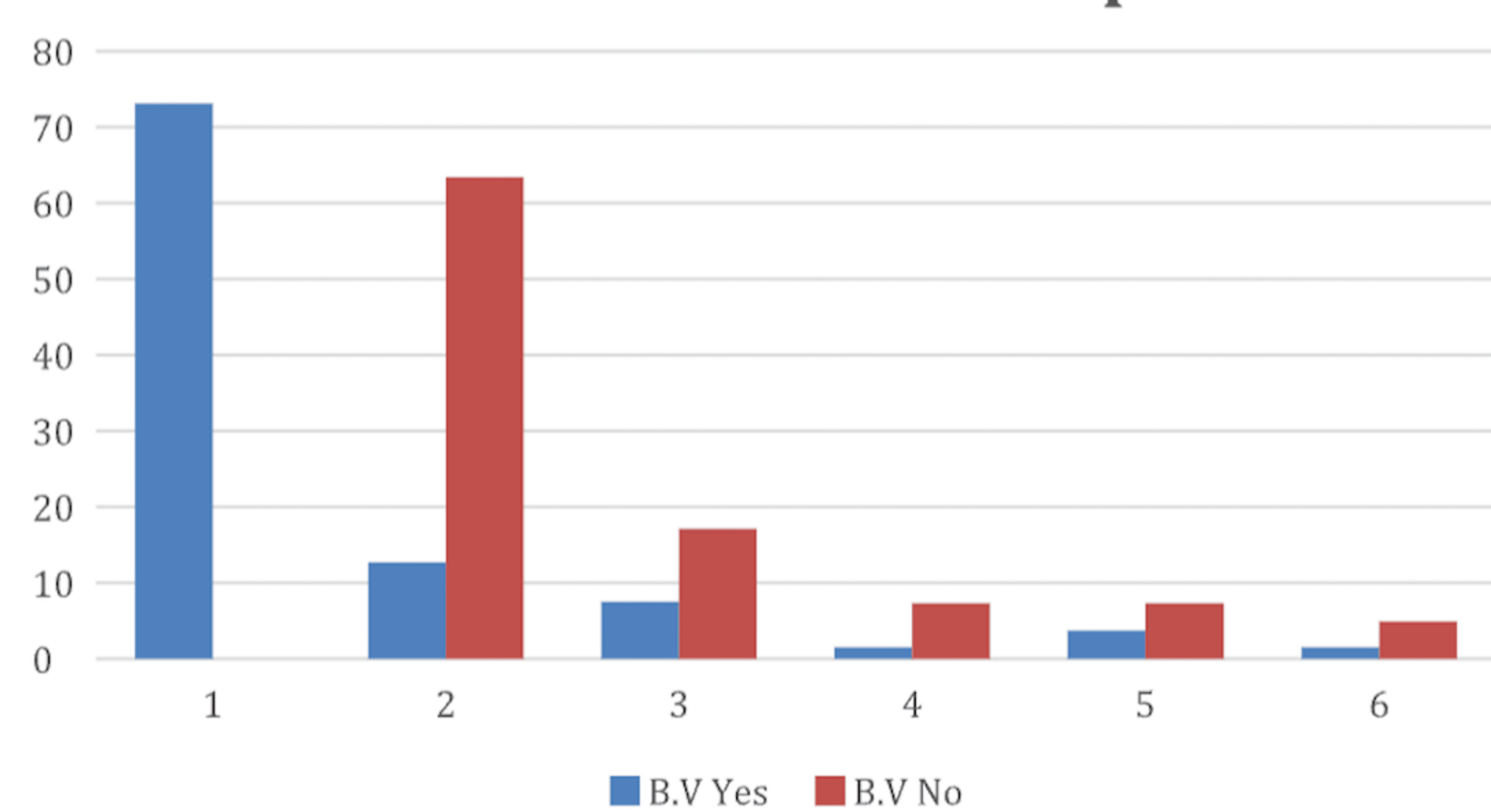
Relationship between STIs and BV STIs, sexually transmitted infections; BV, bacterial vaginosis

Figure 4 illustrates these differences through a bar chart comparison, highlighting the contrasting infection profiles between the two groups. Groups 1-6 depict the types of STIs from Table 3.

Table 4 examines the relationship between race/ethnicity and STI status. Among the 77 individuals who tested positive for an STI, the majority were Hispanic (43), followed by White (17), African American (13), and Pacific Islander (4). Similarly, among the 98 STI-negative individuals, Hispanics again comprised the largest group (64), followed by White (20), African American (12), and Pacific Islander (2). Despite these variations in raw numbers, statistical analysis using the chi-square test revealed no significant association between ethnicity and STI status (χ²=19.8, p=0.7). This suggests that, within this cohort, STI prevalence does not differ significantly by ethnic group.

**Table 4:** Relationship between ethnicity and STIs STIs, sexually transmitted infections

	African American	Hispanic	Pacific Islander	White	Total
STI: Yes	13	43	4	17	77
STI: No	12	64	2	20	98
Total	25	107	6	37	175
	Chi-square 19.8; p=0.707, not significant				

Table 5 explores the distribution of BV status across different ethnic groups. Among the 134 BV-positive individuals, the majority were Hispanic (84), followed by White (27), African American (20), and Pacific Islander (3). In the BV-negative group (n=41), Hispanics still made up a substantial portion (23), along with White (10), African American (5), and Pacific Islander (3) individuals. Despite these differences in raw counts, chi-square analysis revealed no statistically significant association between ethnicity and BV status (χ²=3.015, p=0.38). These findings suggest that BV prevalence in this sample was not significantly influenced by racial or ethnic background.

**Table 5:** Relationship between ethnicity and BV BV, bacterial vaginosis

	African American	Hispanic	Pacific Islander	White	Total
BV: Yes	20	84	3	27	134
BV: No	5	23	3	10	41
Total	25	107	6	37	175
	Chi-square 3.015; p=0.383, not significant				

## Discussion

The findings of this study reveal a clear association between STIs and BV within the sample population. This relationship is supported by biological mechanisms explaining how BV increases susceptibility to STIs. Specifically, the association is due to a shift from an acidic to a more alkaline vaginal pH caused by disruption of vaginal microbiota. Among the two demographic factors examined, ethnicity and age group, a significant correlation emerged between infection status and age. Specifically, the childbearing age group showed a markedly higher prevalence of both STIs and BV, as confirmed by a chi-square analysis (p<0.0001). This highlights a critical vulnerability in this age group, emphasizing the need for targeted education, prevention, and early screening efforts. Increasing awareness and improving access to reproductive health services during these years may help reduce the risk of long-term complications [9].

In contrast, ethnicity did not show a statistically significant association with either STI or BV status. Chi-square analyses for STIs and BV prevalence across ethnic groups resulted in p-values well above the threshold for significance, suggesting that infection risk is distributed relatively equally across ethnicities in this sample. These results underscore the importance of inclusive public health strategies that do not disproportionately target or overlook any one ethnic group. While demographic trends may differ across regions or larger samples, these findings support the idea that prevention and intervention efforts should be equitably applied to all ethnic populations. Untreated, the population is at high risk of sepsis, and if pregnant, the chances of transmitting such life-threatening conditions to the newborn can have disastrous outcomes [10].

Previous literature demonstrates the relationship between BV and STIs. In the journal article "Relationship Between BV and Sexually Transmitted Infections: Coincidence, Consequence or Co-Transmission?," researchers analyzed the prevalence of co-infections involving STI-causing microorganisms and found them significantly more frequent in women with BV than in those without [2]. In this study, 15.2% (44/290) were diagnosed with at least one STI-causing microorganism and 17.2% (50/290) with BV. Furthermore, the prevalence of co-infections involving two STI-causing microorganisms was significantly higher in women with BV than in those without (18% (8/50) vs. 2% (5/250); p<0.001). These findings are strongly reflected in the present study, where a high proportion of individuals were concurrently positive for both BV and STIs. Our data further reinforce the need for integrated screening and prevention strategies targeting women of reproductive age.

Another literature study further demonstrates this strong relationship between BV and one or more STIs. In the journal article "Vaginitis and Risk of Sexually Transmitted Infections: Results of a Multi-Center U.S. Clinical Study Using STI Nucleic Acid Amplification Testing," researchers included a sample of 1051 women diagnosed for the presence or absence of BV and/or symptomatic vulvovaginal candidiasis (VVC). Among this sample, 195 (18.5%) had one or more STIs, including 101 (9.6%) with Trichomonas vaginalis, 24 (2.3%) with Chlamydia trachomatis, 9 (0.8%) with Neisseria gonorrhoeae, and 93 (8.8%) with Mycoplasma genitalium. STI prevalence in BV-positive women was 26.3% (136/518), significantly higher than the 12.5% (59/474) prevalence in BV-negative women (p<0.0002) [11]. This parallels our observation of high concurrent BV and STI rates. Whereas age was a strong predictor in both our sample and the U.S. study, ethnicity did not show a statistically significant effect in our sample. The U.S. clinical study did not report a strong ethnicity effect for BV-STI co-prevalence in its main comparisons. This suggests that age may be a more robust risk factor for BV/STI co-infection than ethnicity, at least in the populations studied.

Despite the valuable insights gained, this study has a few limitations. The sample size was relatively small (n=175) and drawn from a limited timeframe, which may affect the robustness and generalizability of the findings. Furthermore, the sample was predominantly Hispanic (61.1%), potentially introducing demographic bias. On one hand, it offers valuable insights into a population often underrepresented in STIs and BV research, thereby contributing to a more inclusive understanding of health disparities. However, this demographic concentration may limit the extent to which the findings can be generalized to broader, more diverse populations. Cultural, behavioral, and socioeconomic factors that influence STIs and BV risk may differ across racial and ethnic groups, and the patterns observed in this cohort may not fully reflect those in non-Hispanic populations. Moreover, the study did not account for hormonal influences, sexual behavior patterns, socioeconomic factors, or partner data, all of which are relevant to understanding infection risk. These factors were excluded due to constraints in data availability and the scope of the original study design. However, their omission may limit the depth of the conclusions, as hormonal fluctuations can influence vaginal microbiota, behavior patterns affect STI transmission, and socioeconomic factors correlate with healthcare access and risk exposure. Addressing these gaps in future studies could provide a more comprehensive understanding of the complex interplay of biological, behavioral, and social determinants in STIs and BV prevalence.

## Conclusions

In conclusion, this study highlights a significant association between STIs and BV, particularly among individuals of childbearing age. These findings underscore the need to implement targeted educational programs and awareness campaigns focused on this vulnerable demographic. Increasing awareness of coexisting vaginal infections and STIs is a critical step toward decreasing infection rates and improving reproductive health.

From a public health standpoint, it is imperative to expand community health screenings and launch additional initiatives to detect STIs and bacterial vaginosis early. These efforts should be complemented by policies aimed at making healthcare more affordable and accessible to enhance prevention globally. By identifying high-risk groups and prioritizing early intervention, healthcare systems can take meaningful steps toward mitigating reproductive health disparities and adopting a proactive approach to improving outcomes for all women across all age groups and ethnicities.

## References

[1] (2025). Sexually transmitted infections. https://medlineplus.gov/sexuallytransmittedinfections.html.

[2] Abou Chacra L, Ly C, Hammoud A, Iwaza R, Mediannikov O, Bretelle F, Fenollar F (2023). Relationship between bacterial vaginosis and sexually transmitted infections: coincidence, consequence or co-transmission?. Microorganisms.

[3] (2025). Vaginitis and STIs: a complicated web of co-infections. https://www.hologic.com/about/newsroom/vaginitis-and-stis-complicated-web-co-infections.

[4] Ness RB, Hillier S, Richter HE (2003). Can known risk factors explain racial differences in the occurrence of bacterial vaginosis?. J Natl Med Assoc.

[5] World Health Organization. (2025, May 29 (2025). World Health Organization. Sexually transmitted infections (STIs). https://www.who.int/news-room/fact-sheets/detail/sexually-transmitted-infections-(stis).

[6] Mayo Clinic. (2021 (2025). Mayo Clinic. Bacterial vaginosis - symptoms and causes. https://www.mayoclinic.org/diseases-conditions/bacterial-vaginosis/symptoms-causes/syc-20352279.

[7] Allsworth JE, Peipert JF (2011). Severity of bacterial vaginosis and the risk of sexually transmitted infection. Am J Obstet Gynecol.

[8] Gail M Sullivan, Richard Feinn (2025). Power calculator Cohen’s D. National Library of Medicine.

[9] Adler A, Biggs MA, Kaller S, Schroeder R, Ralph L (2023). Changes in the frequency and type of barriers to reproductive health care between 2017 and 2021. JAMA Netw Open.

[10] Chan MY, Smith MA (2017). Infections in pregnancy. Compr Toxicol.

[11] Schwebke JR, Nyirjesy P, Dsouza M, Getman D (2024). Vaginitis and risk of sexually transmitted infections: results of a multi-center U.S. clinical study using STI nucleic acid amplification testing. J Clin Microbiol.

